# Self-reported side effects of tattooing: a cross-sectional study in the Swedish TABOO cohort

**DOI:** 10.1136/bmjph-2026-005480

**Published:** 2026-08-13

**Authors:** Emelie Rietz Liljedahl, Thomas Rustemeyer, Anna Saxne Jöud, Christel Nielsen

**Affiliations:** 1 Department of Laboratory Medicine, Division of Occupational and Environmental Medicine, Lund University Faculty of Medicine, Lund, Sweden; 2 Department of Dermatology and Allergology, Amsterdam University Medical Centres, Amsterdam, The Netherlands; 3 Department of Clinical Sciences Lund, Division of Orthopedics, Lund University Faculty of Medicine, Lund, Sweden; 4 Clinical Pharmacology, Pharmacy and Environmental Medicine, University of Southern Denmark National Institute of Public Health, Odense, Denmark

**Keywords:** Cross-Sectional Studies, Environmental Medicine, Epidemiology, Public Health

## Abstract

**Introduction:**

Tattooing is common in many populations, yet population-based data on side effects remain limited. We aimed to describe the occurrence of self-reported acute- and delayed-onset side effects following tattooing and investigate individual and tattoo-related determinants in a Swedish population.

**Methods:**

The Swedish Tattoo and Body Modifications Cohort includes 13 049 participants, of whom 2633 reported having at least one tattoo and constituted the study sample. In 2021, participants reported predefined acute (within 1 month) and delayed (after 1 month) side effects following tattooing using a structured questionnaire. Information on educational attainment and yearly disposable income was obtained through linkage to national registers. Multivariable logistic regression was used to identify determinants of reporting any acute- and delayed-onset side effects.

**Results:**

Overall, 40% of participants reported at least one acute-onset side effect and 10% reported a delayed-onset side effect following tattooing. A larger tattooed body surface area (>5 hand palms), compared with <1 hand palm, was associated with higher odds of both acute- and delayed-onset side effects (OR 1.81, 95% CI 1.13 to 2.90 and OR 2.06, 95% CI 1.04 to 4.10, respectively). Use of coloured inks, compared with black ink only, was also associated with increased odds of side effects. Acute-onset side effects were associated with younger age, a low-melanin, highly photoreactive skin phenotype, cosmetic tattooing and more tattoo sessions, whereas delayed-onset side effects were most strongly associated with prior acute-onset reactions (OR 8.17, 95% CI 5.45 to 12.20, compared with no acute-onset side effects). Healthcare-seeking for delayed-onset side effects was uncommon.

**Conclusions:**

Acute- and delayed-onset side effects following tattooing were common and associated with individual characteristics and tattoo-related factors. Delayed-onset side effects were strongly associated with prior acute-onset reactions, while healthcare-seeking was uncommon. These findings indicate that a substantial proportion of tattoo-related side effects never reach the healthcare system, highlighting the importance of population-based studies for assessing their occurrence and determinants.

**WHAT IS ALREADY KNOWN ON THIS TOPIC**

- Previous research shows that tattoos are highly prevalent and can lead to reactions, but these effects are incompletely characterised in population-based settings and are likely under-represented in the healthcare system.

**WHAT THIS STUDY ADDS**

- This study provides detailed population-based data on specific tattoo-related symptoms, distinguishing between acute and delayed-onset side-effects. It identifies individual and tattoo-related determinants of side-effects and shows that early reactions strongly predict later complications. We confirmed that most side effects did not lead to healthcare contact, suggesting their true burden is under-reported in healthcare data.

**HOW THIS STUDY MIGHT AFFECT RESEARCH, PRACTICE OR POLICY**

- Together, these findings highlight an under-recognised public health issue and point to gaps in surveillance and risk communication.

## Introduction

 Tattoos have become an increasingly common form of self-expression in many parts of the world. In Sweden, approximately 50% of women under 40 years of age reported having at least one tattoo in 2021.^1^ The age-standardised prevalence in the overall Swedish population is 29%,^1^ and similar proportions have been reported across Western European countries.^2,3^ Given the high prevalence of exposure, tattoo-related health risks—although relatively understudied in population-based settings—may constitute an under-recognised public health concern.

Reactions to tattoos may occur either directly after tattooing (acute-onset) or develop after the wound has healed (delayed-onset). Acute reactions include common inflammatory symptoms such as swelling, redness and itching.^4^ Although these symptoms are often framed as part of the normal healing process, they reflect the physiological response to tissue injury. More severe and often persistent reactions such as granulomatous nodules, blisters, ulceration and scaling may be caused by allergic reactions or by pigment overload, where excessive amounts of pigment are deposited in the dermis to achieve a very dense, opaque colour.^5,6^ Infections can occur if sterile conditions are not maintained during tattooing, leading to symptoms such as fever, delayed wound healing and ulceration.^7^ Photosensitivity of tattooed skin has also been reported,^8^ adding to the range of delayed reactions associated with tattooing.

Despite these documented complications, care-seeking for tattoo-related complaints appears to be uncommon. A German study reported that fewer than 1% of those experiencing tattoo-related side effects sought medical attention.^9^ Instead, people typically turn to their tattooist as the first source of advice, seeking medical attention only for more severe or persistent reactions.^10^ This reliance on tattooists is further illustrated by findings from a US study in which 93% of tattooed individuals perceived tattooists as knowledgeable or very knowledgeable about infectious and allergic complications, compared with 79% and 70% for dermatologists and primary care physicians, respectively.^10^ In addition, in another US study, approximately half of tattooists reported having referred clients to primary care or dermatology for suspected complications.^11^ Collectively, these observations suggest that only a fraction of tattoo-associated reactions are captured within the healthcare system, underscoring the need for population-based studies to obtain accurate estimates of the true occurrence and nature of such side effects.

Population-based surveys from Denmark and Sweden indicated that 5%–10% of tattooed individuals reported side effects, although the specific symptoms were not defined.^2,12^ The Swedish report did not specify the timeframe for symptom onset, whereas the Danish study limited its assessment to reactions occurring later than 3 weeks after tattooing. In a study among 448 French tattooists, 42% reported ever having a reaction, including itch, swelling, itch or swelling after sun exposure, infection or allergy.^13^ A German web-based survey targeting tattooed individuals found that 68% of participants reported acute-onset skin reactions after tattooing, 7% reported acute-onset systemic symptoms and 6% reported persistent problems.^9^ In a US study, 3% of participants reported tattoo-related infections, 4% reported pain in tattooed areas and 23% reported itch within the first month after receiving a tattoo.^10^ Although selection bias may have influenced these estimates, the existing literature nevertheless highlights the need for population-based research to better characterise the occurrence and spectrum of tattoo-related side effects in the general population.

Here, we investigate the occurrence of self-reported side effects in a population-based cohort, distinguishing between acute-onset (within 1 month) and delayed-onset (after 1 month) symptoms. We additionally examine whether the likelihood of reporting such side effects is associated with individual-level and tattoo-related determinants, including sex, age, educational attainment, yearly disposable income, smoking status, skin phenotype, tattoo type (decorative, cosmetic or both), age at first tattoo, tattooed body area, number of tattoo sessions, ink colour and exposure duration. Finally, we assess whether tattooed individuals seek advice for side effects occurring in a healed tattoo and, if so, whether they consult tattooists or healthcare providers.

## Methods

### Study design

We used a cross-sectional study design to investigate self-reported side effects of tattooing in the population-based Swedish Tattoo and Body Modifications Cohort (TABOO). TABOO was established in 2021 and data were collected through self-administered questionnaires assessing tattoo exposure and the experience of any side effects after tattooing.^1^ Information on educational attainment and yearly disposable income was obtained through linkage to national registers.

### Participants

The TABOO cohort has been described in detail elsewhere.^1^ In brief, the cohort was established by pooling the participating controls from three case–control studies assessing tattoo exposure as a potential risk factor for malignant lymphoma, cutaneous melanoma and cutaneous squamous-cell carcinoma among individuals aged 20–60 years.^14–16^ In total, 26 751 controls were invited and 13 049 chose to participate, corresponding to a response rate of 49%. The present analyses were restricted to the participants who reported having at least one tattoo.

### Exposure assessment

In the TABOO cohort, tattoo exposure was defined as the presence or absence of permanent tattoos received for decorative, cosmetic or medical purposes, and participants were asked to include tattoos that had been removed. Individuals with only medical tattoos were excluded from the present analyses.

Information was collected on age at first tattoo, tattooed body surface area (<1 hand palm; 1–5 palms; >5 palms) and the number of tattoo sessions. Participants also reported ink colours, anatomical location, type of tattooist (professional or non-professional) and geographic regions in which the tattoos were received.

### Outcome assessment

Acute-onset side effects, defined as symptoms occurring within 1 month after tattooing, included the following predefined reactions: itch (pruritus), redness, swelling (oedema), wound (ulcer) or scab formation, blisters, papules, fever or shiver, scaling, nodules or callosities, soreness, acute pain, reduced skin sensation, excess scar formation (keloid) and sun sensitivity (photosensitivity).

Delayed-onset side effects, defined as symptoms developing later than 1 month after tattooing, included the same reactions as above, except fever or shiver. Persistent pain was classified as a separate outcome category and defined as pain reported 3 months after tattooing according to the definition of the International Association for the Study of Pain, distinct from pain reported after 1 month.

Participants with multiple tattooing sessions were not asked to specify which session their reported symptoms referred to, implying that each session could contribute a separate at-risk period within the defined time windows.

Respondents were additionally asked whether they sought advice for side effects occurring in a healed tattoo and, if so, whether they consulted a tattooist or a healthcare provider. This question was repeated for each symptom.

### Patient and public involvement

The public—tattooers and volunteers—was involved from the beginning of planning the research, in designing and piloting the study questionnaire. Research questions were not developed and informed by the public’s priorities, experiences or preferences. The public was involved in the conduct of the study by participating, but not in the choice of outcome measures or recruitment to the study. The public will not be involved in choosing methods or agreeing on plans for dissemination of the study results.

### Statistical methods

We used the Phi coefficient to assess the correlations between different symptoms at each time point and visualised them using upset plots. We assessed temporal correlations between acute-onset and delayed-onset reactions for each symptom separately and visualised these relationships using heatmaps.

We used multivariable logistic regression models to investigate individual-level and tattoo-related determinants of reporting any acute- and delayed-onset side effects. There were few individuals with medical tattoos in combination with decorative and/or cosmetic tattoos, and they were therefore excluded from the regression analysis. We used complete-case analysis, as the proportion of missing data was low overall. Variables were selected a priori to investigate socioeconomic and tattoo-related determinants of side effects. All models included sex (female; male), age at survey (continuous), educational attainment (primary and lower secondary; upper secondary; post-secondary), yearly disposable income based on quartiles (<227 kSEK; 227–297 kSEK; 297–374 kSEK; >374 kSEK), smoking (daily; occasionally; previously; never), tattoo type (decorative; cosmetic; both), age at first tattoo (continuous), tattooed body area (<1 palm; 1–5 palms; >5 palms), number of tattoo sessions (1; 2–3; 4–5; 6–9; ≥10) and ink colour scheme (black only; black and colour; colour only). We also included skin phenotype (low melanin, highly photoreactive; low-medium melanin, medium photoreactive; medium-high melanin, low photoreactive according to Rietz Liljedahl *et al*^15^) to account for differences in inflammatory responses across phenotypes.^17^ In the model for delayed-onset side effects, we additionally included a dichotomous variable indicating whether the participant reported any acute-onset side effect (yes; no).

## Results

### Descriptive characteristics

The study sample consisted of the 2633 tattooed individuals who participated in TABOO (20% of the overall cohort). Females were slightly over-represented (61%), and the median age was 53 years (table 1). Most participants had upper-secondary or post-secondary education, whereas only 11% had primary or lower-secondary education. Most participants were ever-smokers (61%), and 11% reported daily smoking at the time of the survey.

**Table 1:** Individual characteristics of the 2633 individuals in the study sample

	n (%) or median (Q1; Q3)
Sex	
Male	1003 (39)
Female	1580 (61)
Age at survey (years)	53 (44; 59)
Educational attainment	
Primary and lower-secondary	279 (11)
Upper-secondary	1363 (53)
Post-secondary	938 (36)
Missing	3
Yearly disposable income (kSEK)	
<227	645 (25)
227–297	646 (25)
297–374	643 (25)
>374	648 (25)
Missing	1
Smoking status	
Daily	296 (11)
Occasional	174 (7)
Former	1098 (43)
Never	1008 (39)
Missing	7
Skin phenotype	
Low melanin, high photoreactivity	710 (28)
Low-medium melanin, medium photoreactivity	1582 (62)
Medium-high melanin, low photoreactivity	273 (11)
Missing	18

The majority of participants (93%) reported having decorative tattoos, while 9% had ever had a cosmetic tattoo (table 2). The median age at first tattoo was 27 years. A tattooed body area of less than one palm was most common (54%), whereas 10% reported tattoos larger than five palms. The most frequently reported ink colour was black, followed by red, blue, green and yellow. Tattoos were primarily received in studios by professional tattooists (83%), and most were obtained in Sweden (87%). Only a small proportion of participants reported having received tattoos outside Europe.

**Table 2:** Tattoo-related characteristics of the 2633 individuals in the study sample

	n (%) or median (Q1; Q3)
Tattoo type*†	
Decorative	2446 (93)
Cosmetic	234 (9)
Medical	22 (1)
Missing	24
Age at first tattoo (years)	27 (19; 40)
Missing	16
Tattooed body area	
<1 palm	1396 (54)
1–5 palms	916 (35)
>5 palms	250 (10)
Missing	21
Number of sessions	
1	1139 (44)
2–3	809 (32)
4–5	291 (11)
6–9	187 (7)
≥10	138 (5)
Missing	19
Colour*	
Black	2161 (83)
Grey	415 (16)
Brown	218 (8)
Red	969 (38)
Blue	716 (28)
Green	732 (28)
Yellow	627 (24)
White	413 (16)
Purple	159 (6)
Pink	192 (7)
Orange	169 (7)
Turquoise	158 (6)
Skin colour (injected)	21 (1)
Other	14 (0.5)
Missing	10
Tattooist and location*	
Professional tattooist, studio	2193 (83)
Professional tattooist, other facility	312 (12)
Cosmetic tattooist, in studio or clinic	185 (7)
Healthcare professional, in clinic	19 (2)
Non-professional, irrespective of location	242 (9)
Missing	20
Geographical region*	
Sweden	2248 (87)
Nordic countries, except Sweden	208 (8)
Rest of Europe	218 (8)
Asia	159 (6)
Oceania	17 (0.7)
USA	59 (2)
Other	59 (2)
Missing	15

* More than one option could be selected.

† Those with only medical tattoos were excluded; the remaining participants had at least one decorative or cosmetic tattoo.

### Side effects

Almost half of the participants (40%) reported having experienced at least one acute-onset side effect following tattooing, while 9% reported a delayed-onset side effect (table 3).

**Table 3:** Occurrence of acute and delayed-onset side effects after tattooing and advice-seeking behaviour among those who reported delayed symptoms

Symptom	Acute-onset n (%)	Delayed-onset n (%)	Advice seeking, tattooist n (% of delayed)	Advice seeking, healthcare n (% of delayed)
Any side effect	1057 (40.1)	237 (9.0)	6 (3)	4 (2)
Inflammatory symptoms			5 (3)*	4 (2)
Itch (pruritus)	432 (16.4)	108 (4.1)		
Redness	482 (18.3)	17 (0.6)		
Swelling (oedema)	381 (14.8)	93 (3.5)		
Soreness	281 (10.7)	10 (0.4)		
Wound (ulcer) or scab formation	577 (22.0)	8 (0.3)		
Other symptoms			3 (4)	0
Blisters	20 (0.8)	3 (0.1)		
Papules	45 (1.7)	26 (1.0)		
Fever or shivering	152 (5.8)	n.a.†		
Scaling	54 (2.1)	5 (0.2)		
Nodules or callosities	3 (0.1)	5 (0.2)		
Pain‡	46 (1.8)	0 (0)		
Reduced skin sensation	15 (0.6)	5 (0.2)		
Excess scar formation (keloid)	9 (0.3)	11 (0.4)		
Photosensitivity	53 (2.1)	55 (2.1)		

* Due to small numbers, individual symptoms were combined into aggregated categories.

† Not assessed.

‡ Pain was also assessed 3 months after tattooing; no participants reported this outcome.

**Figure 1:**
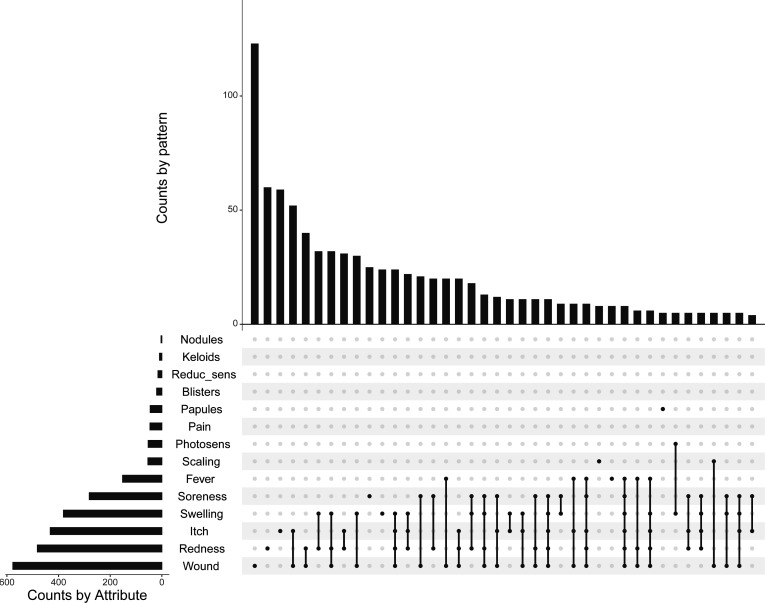
Co-occurrence of acute-onset side effects following tattooing, defined as symptoms occurring after tattooing. Bars show counts per symptom or symptom combination; the matrix indicates included symptoms.

Acute-onset side effects were most commonly inflammatory in nature, including wound or scab formation, redness and itch, reported either individually or in combination, and often together with swelling and soreness (table 3, figure 1). Fever or shivering was reported by almost 6% of participants during the acute period. When acute inflammatory symptoms were considered jointly, they were frequently reported together with fever or shivering, scaling, photosensitivity, bumps (keloids, granulomas, papules or nodules) or pain (online supplemental figure 1).

Acute-onset redness, swelling, itch and wound or scab formation were moderately correlated (ɸ=0.29–0.45, p<0.005; online supplemental figure 2). Fever also showed positive correlations with these acute inflammatory symptoms, although of lower magnitude (ɸ=0.18–0.25, p<0.005). In contrast, correlations involving less common symptoms, such as papules, blisters, scaling, keloids, nodules and reduced skin sensation, were weak or absent.

**Figure 2:**
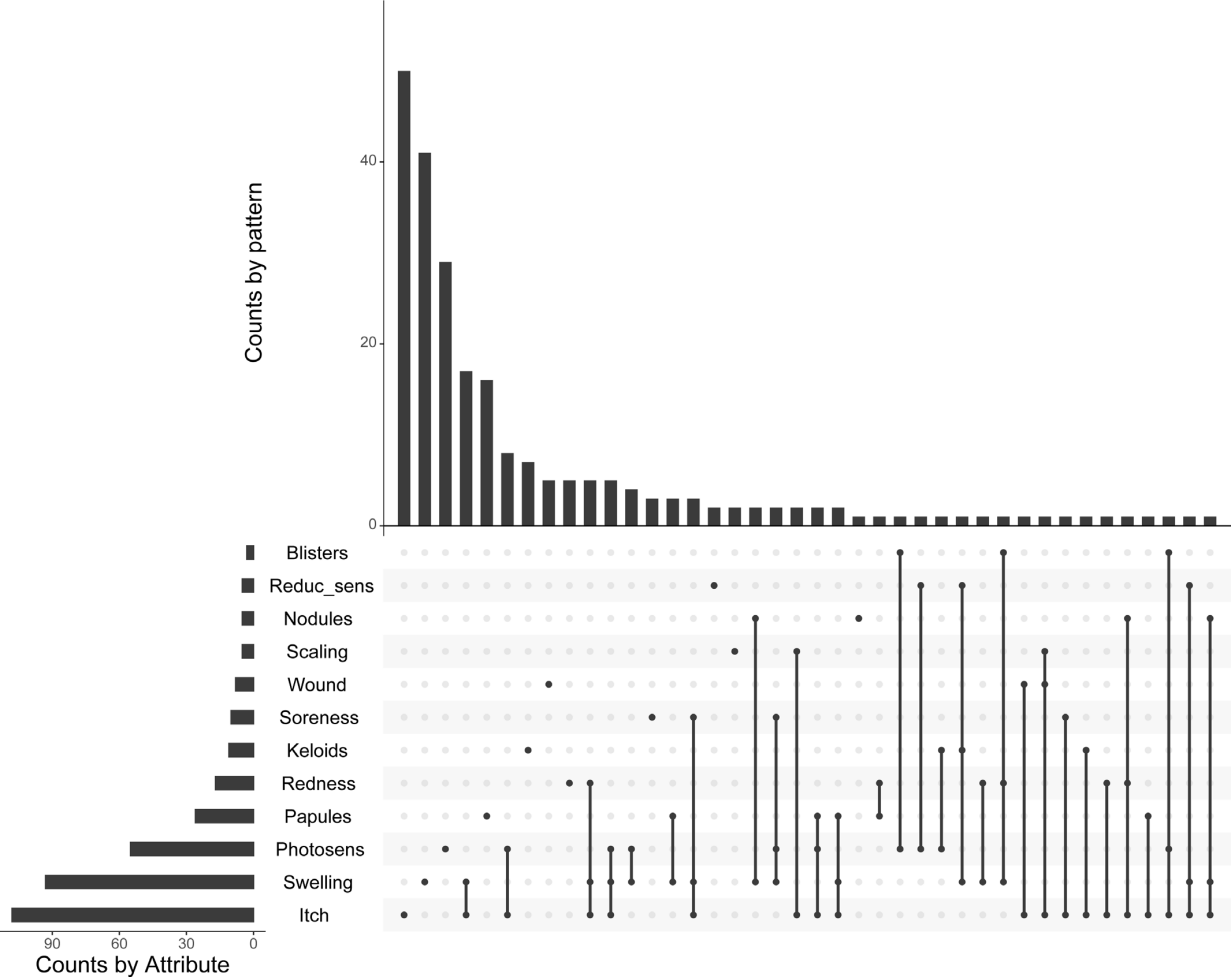
Co-occurrence of delayed-onset side effects following tattooing, defined as symptoms occurring later than 1 month after tattooing. Bars show counts per symptom or symptom combination; the matrix indicates included symptoms.

Delayed-onset side effects were less frequent than acute-onset side effects and most commonly included itch, swelling and photosensitivity (table 3, figure 2). Delayed-onset itch and swelling were moderately correlated (ɸ=0.35, p<0.005; online supplemental figure 3). We observed weak correlations between delayed-onset swelling and redness (ɸ=0.22, p<0.005), itch and redness (ɸ=0.20, p<0.005) and itch and photosensitivity (ɸ=0.20, p<0.005).

Advice-seeking for side effects in healed tattoos was uncommon (table 3). Of the 237 individuals that reported any symptom in a healed tattoo, 96% (n=228) did not seek professional advice. Among the nine individuals who sought advice, consultations with healthcare providers and tattooists occurred in similar proportions (five consulted a tattooist, three consulted a healthcare provider and one individual consulted both).

### Determinants of acute-onset side effects

Being younger at the time of the survey, as well as younger age at first tattoo, were associated with higher odds of reporting any side effect within 1 month of tattooing (table 4). For each additional year of age at the survey, the odds of reporting any acute-onset side effect decreased by 4% (OR=0.96, 95% CI 0.95 to 0.97), while the corresponding decrease was 1% per additional year of age at first tattoo (OR=0.99, 95% CI 0.98 to 1.00). Females appeared to have higher odds of acute-onset symptoms in the univariate analysis, but this association attenuated after adjustment for covariates. Individuals with a skin phenotype characterised by low melanin content had higher odds of reporting acute-onset symptoms compared with those with melanin-rich skin (OR=1.61, 95% CI 1.17 to 2.22). The odds of reporting any acute-onset side effect were higher among individuals with post-secondary educational attainment than among those with primary and lower secondary education, with an OR of 1.87 (95% CI 1.34 to 2.59). We found no associations between yearly disposable income or smoking status and reports of acute-onset side effects.

**Table 4:** Individual-level and tattoo-related determinants of reporting any acute-onset side effect of tattooing (logistic regression)

	Univariate				Multivariable			
	Any symptom (n)	No symptom (n)	OR (95% CI)	P value	Any symptom (n)	No symptom (n)	OR (95% CI)	P value*
Age at survey (years)	1057	1526	0.95 (0.94 to 0.95)	<0.001	1028	1475	0.96 (0.95 to 0.97)	<0.001
Age at first tattoo (years)	1052	1515	0.97 (0.97 to 0.98)	<0.001	1028	1475	0.99 (0.98 to 1.00)	0.035
Sex								0.35
Female	672	908	1.19 (1.01 to 1.40)	0.04	652	880	1.10 (0.90 to 1.34)	0.35
Male	385	618	1.00		376	595	1.00	
Skin phenotype								0.002
Low melanin, high photoreactivity	340	370	1.78 (1.33 to 2.38)	<0.001	335	359	1.61 (1.17 to 2.22)	0.004
Low-medium melanin, medium photoreactivity	616	966	1.23 (0.94 to 1.62)	0.13	605	943	1.19 (0.89 to 1.61)	0.25
Medium-high melanin, low photoreactivity	93	180	1.00		88	173	1.00	
Yearly disposable income (kSEK)								0.85
>374	254	394	0.87 (0.70 to 1.09)	0.23	251	384	0.90 (0.70 to 1.16)	0.41
297–374	269	374	0.97 (0.78 to 1.22)	0.81	263	364	0.95 (0.74 to 1.21)	0.65
227–297	260	386	0.91 (0.73 to 1.14)	0.42	251	378	0.92 (0.72 to 1.17)	0.48
<227	274	371	1.00		263	349	1.00	
Smoking status								0.23
Daily	105	191	0.77 (0.59 to 1.01)	0.06	100	184	0.85 (0.63 to 1.16)	0.31
Occasional	84	90	1.31 (0.95 to 1.80)	0.10	80	84	1.20 (0.83 to 1.72)	0.34
Former	446	652	0.96 (0.81 to 1.14)	0.63	437	632	1.12 (0.92 to 1.36)	0.25
Never	420	588	1.00		411	575	1.00	
Educational attainment								<0.001
Post-secondary	458	480	2.18 (1.64 to 2.90)	<0.001	445	471	1.87 (1.34 to 2.59)	<0.001
Upper secondary	514	849	1.38 (1.05 to 1.82)	0.02	502	820	1.26 (0.93 to 1.71)	0.14
Primary and lower secondary	85	194	1.00		81	184	1.00	
Tattoo type								0.005
Decorative and cosmetic	64	57	1.68 (1.17 to 2.43)	0.006	62	55	1.00 (0.66 to 1.53)	0.97
Cosmetic	55	58	1.42 (0.97 to 2.07)	0.07	53	55	2.09 (1.34 to 3.26)	0.001
Decorative	931	1394	1.00		913	1365	1.00	
Colour scheme								<0.001
Black and colour	590	636	1.75 (1.47 to 2.07)	<0.001	574	617	1.67 (1.36 to 2.05)	<0.001
Colour only	122	234	0.98 (0.76 to 1.27)	0.88	117	222	1.53 (1.14 to 2.06)	0.005
Black only	344	647	1.00		337	636	1.00	
Tattooed body area								0.04
>5 palms	172	78	4.44 (3.33 to 5.94)	<0.001	166	75	1.81 (1.13 to 2.90)	0.01
1–5 palms	416	500	1.68 (1.41 to 1.99)	<0.001	410	493	1.18 (0.94 to 1.48)	0.16
<1 palm	463	933	1.00		452	907	1.00	
Number of sessions								0.002
≥10	96	42	5.37 (3.66 to 7.89)	<0.001	94	41	2.15 (1.21 to 3.84)	0.009
6–9	114	73	3.67 (2.66 to 5.05)	<0.001	113	73	1.97 (1.29 to 3.02)	0.002
4–5	145	146	2.33 (1.80 to 3.03)	<0.001	139	138	1.58 (1.13 to 2.21)	0.008
2–3	355	454	1.84 (1.52 to 2.22)	<0.001	351	449	1.51 (1.21 to 1.88)	<0.001
1	340	799	1.00		331	774	1.00	

* For continuous variables, p values test the null hypothesis that the regression coefficient equals zero. For categorical variables, overall p values test the joint null hypothesis of no association (ie, that all category coefficients equal zero), whereas category-specific p values compare each category with the reference group.

Among the tattoo-related determinants, cosmetic tattoos were associated with doubled odds of reporting acute-onset side effects in relation to decorative tattoos (OR=2.09, 95% CI 1.34 to 3.26). A colour scheme containing both black and colourful inks was also associated with elevated odds of acute-onset symptoms (OR=1.67, 95% CI 1.36 to 2.05), as was a colour scheme of only colourful inks (OR=1.53, 95% CI 1.14 to 2.06), compared with having tattoos with black ink only. Finally, a tattooed body surface larger than five hand palms (OR=1.81, 95% CI 1.13 to 2.90, compared with less than one palm) and a higher number of tattoo sessions (OR=2.15, 95% CI 1.21 to 3.84, for 10 sessions or more, compared with one session), were associated with higher odds of having experienced any side effect within 1 month of tattooing.

### Determinants of delayed-onset side effects

The primary determinant of reporting delayed-onset side effects was having experienced acute-onset side effects (table 5). Individuals who reported any side effect within 1 month of tattooing had eightfold higher odds of also experiencing delayed-onset side effects (OR=8.17, 95% CI 5.45 to 12.20).

**Table 5:** Individual-level and tattoo-related determinants of reporting any delayed-onset side effect of tattooing (logistic regression)

	Univariate				Multivariable			
	Any symptom (n)	No reaction (n)	OR (95% CI)	P value	Any symptom (n)	No reaction (n)	OR (95% CI)	P value*
Any acute-onset side effect†								<0.001
Yes	205	852	11.00 (7.70 to 16.00)	<0.001	197	831	8.17 (5.45 to 12.2)	<0.001
No	32	1494	1.00		31	1444	1.00	
Age at survey (years)	237	2346	0.95 (0.94 to 0.96)	<0.001	228	2275	0.98 (0.97 to 1.01)	0.07
Age at first tattoo (years)	234	2333	0.95 (0.94 to 0.97)	<0.001	228	2275	0.97 (0.96 to 0.99)	0.003
Sex								0.11
Female	154	1426	1.20 (0.91 to 1.58)	0.21	149	1383	1.33 (0.94 to 1.88)	0.11
Male	83	920	1.00		79	892	1.00	
Skin phenotype								0.10
Low melanin, high photoreactivity	82	628	1.19 (0.75 to 1.88)	0.27	82	612	0.87 (0.51 to 1.47)	0.60
Low-medium melanin, medium photoreactivity	125	1457	0.78 (0.51 to 1.21)	0.46	121	1427	0.66 (0.40 to 1.08)	0.10
Medium-high melanin, low photoreactivity	27	246			25	236	1.00	
Yearly disposable income (kSEK)								0.88
>374	50	598	0.69 (0.47 to 1.01)	0.05	49	586	0.90 (0.58 to 1.39)	0.63
297–374	660	583	0.85 (0.59 to 1.22)	0.37	58	569	0.93 (0.62 to 1.41)	0.75
227–297	57	589	0.80 (0.55 to 1.15)	0.22	55	574	0.85 (0.56 to 1.28)	0.42
<227	70	575	1.00		66	546	1.00	
Smoking status								0.01
Daily	19	277	0.87 (0.51 to 1.46)	0.59	18	266	0.83 (0.46 to 1.49)	0.53
Occasional	25	149	2.12 (1.30 to 3.44)	0.002	24	140	1.71 (0.99 to 2.97)	0.06
Former	117	981	1.51 (1.11 to 2.04)	0.009	114	955	1.56 (1.11 to 2.19)	0.01
Never	74	934	1.00		72	914	1.00	
Educational attainment								0.28
Post-secondary	97	841	1.90 (1.10 to 3.28)	0.02	94	822	1.60 (0.83 to 3.08)	0.16
Upper secondary	124	1239	1.65 (0.96 to 2.82)	0.07	121	1201	1.67 (0.89 to 3.13)	0.11
Primary and lower secondary	16	263	1.00		13	252	1.00	
Tattoo type								0.10
Decorative and cosmetic	23	98	2.40 (1.49 to 3.87)	<0.001	22	95	1.79 (1.03 to 3.13)	0.04
Cosmetic	6	107	0.57 (0.25 to 1.32)	0.19	5	103	0.80 (0.29 to 2.22)	0.67
Decorative	207	2118	1.00		201	2077	1.00	
Colour scheme								0.005
Black and colour	154	1072	2.27 (1.66 to 3.10)	<0.001	150	1041	1.83 (1.27 to 2.64)	0.001
Colour only	24	332	1.14 (0.70 to 1.87)	0.60	21	318	1.70 (0.93 to 3.10)	0.09
Black only	59	932	1.00		57	916	1.00	
Tattooed body area								0.08
>5 palms	48	202	3.91 (2.66 to 5.76)	<0.001	48	193	2.06 (1.04 to 4.10)	0.04
1–5 palms	107	809	2.18 (1.61 to 2.95)	<0.001	104	799	1.54 (1.00 to 2.39)	0.05
<1 palm	80	1316	1.00		76	1283	1.00	
Number of sessions								0.17
≥10	21	117	3.02 (1.78 to 5.12)	<0.001	21	114	0.50 (0.22 to 1.18)	0.11
6–9	38	149	4.29 (2.77 to 6.63)	<0.001	38	148	1.03 (0.53 to 2.01)	0.92
4–5	43	248	2.91 (1.93 to 4.39)	<0.001	42	235	1.15 (0.65 to 2.03)	0.64
2–3	68	741	1.54 (1.08 to 2.20)	0.02	67	733	0.87 (0.56 to 1.36)	0.54
1	64	1075	1.00		60	1045	1.00	

* For continuous variables, p values test the null hypothesis that the regression coefficient equals zero. For categorical variables, overall p values test the joint null hypothesis of no association (ie, that all category coefficients equal zero), whereas category-specific p values compare each category with the reference group.

† Defined as symptoms occurring within 1 month of tattooing.

As for acute-onset symptoms, younger age at first tattoo was associated with higher odds of reporting side effects occurring later than 1 month after tattooing, with the odds decreasing by 3% for each additional year of age at first tattoo (OR=0.97, 95% CI 0.96 to 0.99). Female sex was associated with higher odds of reporting delayed-onset side effects, although the estimate was imprecise. Individuals with a melanin-rich skin phenotype had higher odds of reporting delayed-onset symptoms compared with those with low or low-medium melanin skin phenotypes, as reflected by lower point estimates among the latter groups (OR=0.87, 95% CI 0.51 to 1.47 and OR=0.66, 95% CI 0.40 to 1.08, respectively). Occasional smokers (OR=1.71, 95% CI 0.99 to 2.97) and former smokers (OR=1.56, 95% CI 1.11 to 2.19) had higher odds of reporting delayed-onset side effects than never smokers. Compared with individuals with primary or lower secondary education, those with post-secondary or upper secondary education had higher odds of reporting delayed-onset symptoms, although with low precision. Yearly disposable income was not associated with the odds of reporting delayed-onset side effects.

Individuals with both decorative and cosmetic tattoos had higher odds of reporting delayed-onset symptoms than those with decorative tattoos only (OR=1.79, 95% CI 1.03 to 3.13). Individuals with cosmetic tattoos only, although few, did not have higher odds of delayed-onset symptoms. Similar to acute-onset symptoms, higher odds of delayed-onset side effects were observed among individuals with tattoos containing both black and coloured inks (OR=1.83, 95% CI 1.27 to 2.64) and among those with coloured inks only, although the latter estimate was imprecise (OR=1.70, 95% CI 0.93 to 3.10), with black ink only as the reference. A larger tattooed body surface was monotonically associated with higher odds of delayed-onset side effects. Individuals with a tattooed body surface of one to five hand palms had 53% higher odds of delayed-onset side effects (OR=1.54, 95% CI 1.00 to 2.39), while those with a tattooed body surface of five hand palms or more had more than doubled odds (OR=2.06, 95% CI 1.04 to 4.10), compared with individuals with a tattooed body area of less than one hand palm. In contrast, no corresponding association was observed for the number of tattoo sessions.

## Discussion

In this population-based study, we found that self-reported side effects after tattooing were common, with 40% of tattooed individuals reporting at least one acute-onset side effect and 10% reporting a delayed-onset side effect. Both acute- and delayed-onset reactions involved inflammatory symptoms, although their clinical presentation differed. Acute-onset reactions were predominantly characterised by inflammatory wound-healing symptoms (redness, swelling, itch, wound or scab formation), whereas delayed-onset symptoms occurred in healed skin and most commonly included itch, swelling and photosensitivity. Despite the relatively high occurrence of delayed-onset side effects, only four participants sought healthcare. A larger tattooed body surface and use of coloured ink emerged as common determinants of both acute- and delayed-onset side effects. Acute-onset side effects were additionally associated with younger age, a low-melanin, highly photoreactive skin phenotype, post-secondary educational attainment, cosmetic tattooing and a higher number of tattoo sessions. Delayed-onset side effects were most strongly associated with prior acute-onset reactions but were also associated with younger age at first tattoo, smoking history and combined decorative and cosmetic tattoos.

The reported occurrence of tattoo-related side effects in the published literature varies widely.^2,9,10,12,13^ Among studies that defined the timing of symptoms, Klügl *et al*^9^ reported that 68% of participants experienced skin-related side effects and 7% reported systemic symptoms directly after tattooing, which is comparable to the occurrence of acute-onset symptoms observed in the present study. Similarly, Liszewski *et al*^10^ reported that early tattoo-related complaints, including itch and pain, were common within the first month after tattooing. Consistent with this pattern, we observed moderate correlations among acute-onset inflammatory symptoms, including redness, swelling, soreness or pain, itch and wound formation. Together, these findings indicate that acute inflammatory reactions are frequently reported following tattoo exposure.

Side effects related to inflammation and wound healing are often regarded as part of normal tattoo healing and reflect physiological responses to repeated skin trauma.^4^ However, inflammation represents an activated immune state and repeated or pronounced inflammatory responses should not be considered entirely benign, as they may have implications beyond the immediate healing phase. Distinguishing transient inflammatory responses to tissue trauma from reactions that persist or arise after healing is therefore important.

In the present study, 10% of participants reported delayed-onset side effects, which is comparable to the occurrence of skin-related side effects more than 3 weeks after tattooing in a population-based Danish study.^2^ Delayed-onset itch and swelling were the most frequently reported symptoms after healing and showed moderate correlations with each other. Unlike early inflammatory responses related to tissue trauma, these symptoms are unlikely to reflect transient wound-healing processes and may instead indicate ongoing low-grade inflammatory activity or altered immune reactivity in tattooed skin.^18,19^

Although uncommon in our population-based sample, the occurrence of papules or nodules together with delayed-onset inflammatory symptoms may be clinically relevant. Such symptom constellations have previously been described in association with allergic or granulomatous reactions to tattoo pigments, including sarcoidosis,^20,21^ and may therefore represent manifestations of immune-mediated processes rather than benign late reactions.

While the clinical significance of delayed-onset symptom patterns remains unclear, their occurrence after healing suggests that such symptoms may be informative markers of altered immune reactivity following tattooing. Whether these patterns are associated with subsequent immune-mediated disease cannot be addressed in the present cross-sectional study and warrants investigation in longitudinal settings.

Several biological mechanisms may plausibly underlie the observed differences between acute- and delayed-onset side effects. Tattooing involves repeated penetration and injection of ink into the dermis, causing tissue injury and triggering an acute inflammatory response aimed at wound healing. This process is characterised by vasodilation, infiltration of innate immune cells and the release of pro-inflammatory mediators, which likely explains the frequent co-occurrence of redness, swelling, soreness, itch and wound formation observed shortly after tattooing.^4^ In most individuals, this acute inflammatory response is transient and resolves as tissue integrity is restored, usually within 1 month. Our definitions of acute and delayed-onset side effects were chosen to distinguish between such expected reactions during the healing period and reactions developing with delayed onset, but it should be acknowledged that this is a pragmatic cut-off and different time thresholds could have been justified.

Tattoo inks are recognised as exogenous substances by local immune cells and are phagocytosed by dermal macrophages, as shown in mural models,^18,22^ with available evidence suggesting subsequent transport of pigment particles to regional lymph nodes.^23,24^ While this process contributes to the long-term persistence of tattoos, the fate of tattoo pigments beyond lymphatic translocation remains largely unknown. In some individuals, the combination of repeated tissue injury and persistent antigenic stimulation from deposited pigments may result in sustained or recurrent immune activation rather than resolution of inflammation.

Traumatic skin injury can, in certain circumstances, trigger exaggerated or prolonged inflammatory responses, either directly or through repeated inflammatory episodes.^25^ If inflammatory triggers cannot be adequately resolved and pro-inflammatory signalling persists, immune homeostasis may be disrupted, potentially leading to a state of chronic low-grade inflammation.^26^ Such chronic inflammatory states have been implicated in the development of immune-mediated and other non-communicable diseases.^27^ In the context of tattooing, acute- and delayed-onset side effects may therefore reflect different manifestations along a continuum of immune activation, although this hypothesis could not be evaluated with the present study design.

In line with this reasoning, if delayed-onset symptoms reflect sustained immune activation, factors that increase tissue injury, antigenic load or immune stimulation would be expected to increase risk. In the present study, both acute- and delayed-onset side effects were more frequently reported among individuals with larger tattooed body surface areas, supporting the notion that cumulative exposure may be relevant for both early and later side effects. In contrast, while a higher number of tattoo sessions was associated with an increased odds of acute-onset side effects, no clear association was observed for delayed-onset side effects.

The association between a higher number of tattoo sessions and acute-onset side effects is biologically plausible, as repeated sessions may result in repetitive triggering of the innate immune system, resulting in short-term inflammatory responses. In contrast, the absence of an association between session count and delayed-onset side effects may partly reflect selection processes and heterogeneity in tattooing behaviour, whereby individuals who experience reactions early may be less likely to undergo additional sessions. Moreover, the number of sessions does not fully capture cumulative exposure, as sessions may vary in temporal spacing and tattooed area. In this context, tattooed body surface area—used as a pragmatic proxy for cumulative tattoo exposure in the limited existing literature - may capture aspects of long-term antigenic load not fully reflected by session count alone. However, as these measures are correlated, their independent effects are difficult to disentangle.

Ink composition and pigment diversity also emerged as relevant determinants of tattoo-related side effects. Red ink is frequently highlighted as particularly reactive, and in the present study tattoos containing coloured ink in combination with black ink were associated with a higher likelihood of reported side effects compared with tattoos using black ink only. A plausible explanation is that exposure to a broader range of pigments increases the diversity of chemical compounds introduced into the skin, thereby increasing antigenic complexity and the potential for immune stimulation. The immune system may therefore be more likely to respond when exposed to multiple distinct compounds rather than a more uniform pigment composition.

Cosmetic tattooing was similarly associated with a higher risk of acute-onset side effects compared with decorative tattooing. Cosmetic tattoos often contain red or brown pigments and may include contaminants such as aromatic amines, as documented in regulatory surveillance.^28^ While it is not possible to disentangle the relative contributions of specific pigments, contaminants and mechanical tissue injury in the present study, these findings are consistent with the hypothesis that pigment composition and chemical complexity influence the likelihood of inflammatory reactions following tattooing.

Individual-level characteristics also influenced the likelihood of reporting tattoo-related side effects. Although a Danish population-based study reported a higher frequency of tattoo reactions among women,^2^ we did not observe a corresponding association after adjustment for other risk factors, indicating that sex does not appear to contribute independently to the prediction of tattoo-related side effects once other correlated determinants are taken into account. Increasing age, both at survey response and at first tattooing, was consistently associated with a lower likelihood of reported side effects, which may reflect age-related changes in immune responsiveness.^29^

Differences in inflammatory responses related to skin melanin content have been described in the literature.^17^ In the present study, individuals with lower melanin content had a higher likelihood of acute-onset side effects, whereas a tendency toward a lower risk of delayed-onset side effects was observed compared with individuals with higher melanin content. This pattern is consistent with reports suggesting that inflammatory responses may be more readily apparent in low-melanin skin, while melanin-rich skin may be more prone to low-grade chronic inflammatory activity.^17^ Together, these findings suggest that susceptibility to tattoo-related side effects may vary across demographic and phenotypic characteristics, influencing the biological expression of inflammatory responses as well as their clinical or phenotypic presentation.

In this population-based sample, only a very small proportion of individuals reporting delayed-onset symptoms sought advice from healthcare providers, indicating that many tattoo-related side effects are managed outside formal medical care or not addressed at all. This pattern is consistent with previous studies, which have similarly reported low levels of healthcare contact for tattoo-related complaints and a reliance on tattooists or self-management rather than medical consultation.^2,9,10^ Limited healthcare-seeking behaviour implies that tattoo-related side effects are unlikely to be captured in medical records or registers, with important implications for both clinical recognition and epidemiological assessment.

Health-seeking behaviour is socially patterned and more common among individuals with higher socioeconomic status. Higher socioeconomic status may therefore be associated with both a greater likelihood of also reporting tattoo-related side effects, which could help explain why higher socioeconomic status appeared to be associated with higher odds of reporting tattoo-related side effects in this study. Conversely, side effects among individuals with lower socioeconomic status may be systematically under-reported. However, the average monthly disposable income in our sample was slightly lower than that of the overall Swedish population (26 740 SEK vs 27 725 SEK in 2021^30^), indicating that the occurrence of tattoo-related side effects observed in this study is likely an underestimate.

Given the current state of knowledge, it is not possible to disentangle the relative contributions of ink composition and physical tissue injury to the occurrence of tattoo-related side effects. This uncertainty is directly relevant for the interpretation of the present findings, as evidence indicates incomplete regulatory compliance in the tattoo ink market. The Swedish Medical Products Agency recently identified prohibited substances, or concentrations exceeding permitted limits, in a random sample of tattoo inks purchased from various online marketplaces.^28^ Similarly, a recent study reported undeclared ingredients in tattoo inks from the USA, where corresponding regulation is lacking.^31^ Taken together, this body of evidence places the observed associations in the present study in a broader regulatory context, where compliance and transparency in tattoo ink composition remain important for exposure assessment.

### Strengths and limitations

A major strength of this study is the population-based design of the TABOO cohort, which enables assessment of tattoo-related side effects beyond those that come to clinical attention. This is particularly important given that many reactions are mild, transient or managed outside formal healthcare. Another key strength is the detailed characterisation of self-reported side effects, including a clear distinction between acute- and delayed-onset reactions and a broad range of predefined symptoms, allowing analyses of symptom patterns rather than aggregated outcomes. The study also benefits from rich exposure information, including tattoo type, tattooed body surface area, number of sessions, ink colour, enabling a more nuanced assessment of tattoo exposure than binary classifications, as well as linkage to register-based data on education and income.

Several limitations should be acknowledged. First, a cross-sectional design usually limits the ability to examine how determinants relate to the development and persistence of outcomes over time. However, unlike many cross-sectional studies that rely on prevalent outcome measures, we specifically captured incident side effects because of how the survey questions were designed. This temporal ordering reduces concerns regarding reverse causation. Furthermore, because the outcomes were non-disabling or life-threatening, the risk of selective participation or censoring due to the outcome itself is likely to be low. Consequently, some of the limitations commonly associated with cross-sectional studies based on prevalent outcome measures are likely less of a concern in the present study. Second, all data were self-reported, which may introduce misclassification, particularly for tattoos received many years before the survey. Furthermore, the association between acute- and delayed-onset side effects may be influenced by residual confounding due to differences in symptom reporting behaviour beyond the socioeconomic factors accounted for.

However, the overall occurrence of tattoo-related side effects observed in the present study was comparable to that reported in a recent population-based Danish study, suggesting that substantial over-reporting is unlikely. Finally, the study lacked detailed information on ink composition and pigment concentrations, which is difficult to obtain in large-scale observational studies, limiting the ability to link reported side effects to specific chemical constituents. In addition, information on the temporal spacing between tattoo sessions was not collected, precluding assessment of how the timing of repeated exposures may influence the dynamics of inflammatory responses following tattooing.

## Conclusion

Both acute- and delayed-onset side effects following tattooing were common in this population, particularly among individuals with larger tattooed body surface areas, cosmetic tattoos and tattoos involving both black and coloured inks. Despite the relatively high occurrence of symptoms, healthcare-seeking for tattoo-related side effects was uncommon. These findings indicate that a substantial proportion of tattoo-related reactions occur outside the healthcare system and are therefore unlikely to be captured in medical records. Population-based studies are thus essential for assessing the occurrence and determinants of tattoo-related side effects and for understanding their public health relevance.

## Supplementary material

10.1136/bmjph-2026-005480 online supplemental file 1

## Data Availability

Data are available upon reasonable request.
