# Supplementary material for: Self-reported side effects of tattooing: a cross-sectional study in the Swedish TABOO cohort
Source: BMJ Public Health. 2026 Aug 13;4(3):e005480. doi: 10.1136/bmjph-2026-005480 (PMC13475460; doi:10.1136/bmjph-2026-005480)
Supplement: Supplementary file 1 [file bmjph-4-3-s001.docx]

# Supplementary material to “Self-reported side effects of tattooing – A study in the Swedish TABOO cohort”

Emelie Rietz Liljedahl^1^, Thomas Rustemeyer^2^, Anna Saxne Jöud^1,3^, Christel Nielsen^1,4^

^1^Division of Occupational and Environmental Medicine, Department of Laboratory Medicine, Lund University, Lund, Sweden

^2^Department of Dermatology and Allergology, Amsterdam University Medical Center, Amsterdam, The Netherlands

^3^Division of Orthopedics, Department of Clinical Sciences Lund, Lund University, Lund, Sweden

^4^Clinical Pharmacology, Pharmacy and Environmental Medicine, Institute of Public Health, University of Southern Denmark, Odense, Denmark

Supplementary figure 1. Acute self-reported symptoms sorted by counts of individual items and counts of patterns of items reported together, with inflammatory symptoms and symptoms representing bumps grouped.

Supplementary figure 2. Correlation matrix of acute-onset side effects following tattooing, defined as defined as symptoms occurring within one month after tattooing.
*Photosensitivity, reduced sensitivity.

Supplementary figure 3. Correlation matrix of delayed-onset side effects following tattooing, defined as symptoms occurring later than one month of tattooing. *Photosensitivity, reduced sensitivity.
